# Empirical and model-based estimates of spatial and temporal variations in net primary productivity in semi-arid grasslands of Northern China

**DOI:** 10.1371/journal.pone.0187678

**Published:** 2017-11-07

**Authors:** Shengwei Zhang, Rui Zhang, Tingxi Liu, Xin Song, Mark A. Adams

**Affiliations:** 1 College of Water Conservancy and Civil Engineering, Inner Mongolia Agricultural University, Hohhot, China; 2 Centre for Carbon, Water and Food, University of Sydney, Sydney, Australia; 3 College of Life Sciences and Oceanography, Shenzhen University, Shenzhen, China; 4 Swinburne University of Technology, Faculty of Science Engineering and Technology, Hawthorn, Victoria, Australia; Pacific Northwest National Laboratory, UNITED STATES

## Abstract

Spatiotemporal variations in net primary productivity (NPP) reflect the dynamics of water and carbon in the biosphere, and are often closely related to temperature and precipitation. We used the ecosystem model known as the Carnegie-Ames-Stanford Approach (CASA) to estimate NPP of semiarid grassland in northern China counties between 2001 and 2013. Model estimates were strongly linearly correlated with observed values from different counties (slope = 0.76 (p < 0.001), intercept = 34.7 (p < 0.01), R^2^ = 0.67, RMSE = 35 g C·m^-2^·year^-1^, bias = -0.11 g C·m^-2^·year^-1^). We also quantified inter-annual changes in NPP over the 13-year study period. NPP varied between 141 and 313 g C·m^-2^·year^-1^, with a mean of 240 g C·m^-2^·year^-1^. NPP increased from west to east each year, and mean precipitation in each county was significantly positively correlated with NPP—annually, and in summer and autumn. Mean precipitation was positively related to NPP in spring, but not significantly so. Annual and summer temperatures were mostly negatively correlated with NPP, but temperature was positively correlated with spring and autumn NPP. Spatial correlation and partial correlation analyses at the pixel scale confirmed precipitation is a major driver of NPP. Temperature was negatively correlated with NPP in 99% of the regions at the annual scale, but after removing the effect of precipitation, temperature was positively correlated with the NPP in 77% of the regions. Our data show that temperature effects on production depend heavily on recent precipitation. Results reported here have significant and far-reaching implications for natural resource management, given the enormous size of these grasslands and the numbers of people dependent on them.

## Introduction

Net primary productivity (NPP), or the increment (on a daily to yearly basis) in the amount of carbon stored on land or in the oceans, is the balance between photosynthesis and respiration [1]. NPP thus reflects the net carbon input from the atmosphere to either the terrestrial or marine biospheres [2]. Relations of NPP to basic climatic conditions can provide a basis for theoretical and practical predictions of local-to-global carbon cycles [3,4]. Derived relationships can also provide guidance for sustainable use of resources and realization of the productive potential of ecosystems [5–7,1]. Grasslands are important components of many terrestrial ecosystems [8] and are widely studied due to their sensitivity to changing climates [9–11]. Process models have been used to explore underlying biological processes in grasslands (such as photosynthesis, respiration and transpiration) as well as mechanisms driving interactions between these processes and environmental parameters [12,13].

In recent years, remote sensing data have been used in conjunction with models to estimate regional and global NPP [14–17]. For example, the Carnegie-Ames-Stanford Approach (CASA) has been used to estimate changes in the NPP of vegetation [18–20] as well light use efficiency (via photosynthetically active radiation) [21]. A range of remotely sensed data provide inputs for CASA models. For example, thematic mapper (TM) [22] provides surface distribution of NPP at a relatively high spatial resolution, while the advanced very-high-resolution radiometer (AVHRR) [23], and moderate resolution imaging spectroradiometer (MODIS) [15,24] provide further data that can be used to help predict NPP over large areas.

Climate (temperature, precipitation, etc.) exerts critical control of vegetation growth in most ecosystems [10,25,26] and relationships between NPP and climate are widely studied using different methods [13,27]. For example, linear regression and covariance have been used to assess relationships between aboveground NPP (ANPP) and temperature and precipitation (annually and during the growing season) [3]. For grassland ecosystems in Inner Mongolia, Zhang et al. [28] estimated the spatial distribution of NPP in the Balager River Basin of the Xilingol Grassland using a light use efficiency model and analysed correlations among climate factors, vegetation indices and NPP. They found that precipitation and monthly mean temperature both correlated well with NPP and that precipitation had a greater impact than temperature [28]. Mu et al. [29] used remote sensing of the vegetation and the CASA model to reveal spatiotemporal dynamics of NPP for different types of vegetation as well as their differences in NPP responses to climate. Zhang et al. [30] used the CENTURY model to simulate changes in the ANPP of grasslands in Xilinhot and their responses to climate change over the past 58 years. They showed that the ANPP of typical Inner Mongolian grasslands was highly sensitive to climate change, with distinct variation due to changes in temperature and precipitation. Gao et al. [31] found that NPP of semiarid grasslands of Inner Mongolia were significantly affected by biomass allocation and precipitation use efficiency. Management impacts on NPP have also been analysed [32]. Lkhagva et al. [33] found that excessive grazing reduced the distribution of bryophytic vegetation, and encouraged the disappearance of frozen soils, and climate warming.

Most previous studies have focused on NPP and its relationship to climate at either annual scales, or during the growing season. We investigated these relationships at different scales, and the synergistic interactions among climatic variables. We assessed the NPP dynamics of a semiarid grassland (i.e., the Xilingol Grassland) between 2001 and 2013 using a light use efficiency model in combination with spatial and temporal data. Correlations and partial correlations between NPP and precipitation and temperature were analysed spatially at the pixel level, and annual and seasonal temporal scales.

## Materials and methods

### General study area information

**Fig 1:**
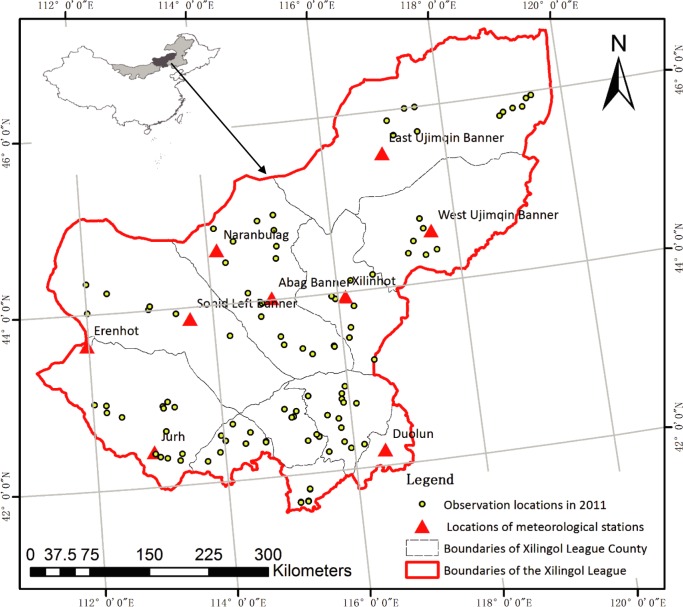
Study area and locations of meteorological stations and observation locations in 2011.

The Xilingol Grassland (115°13'–117°06'E and 43°02'–44°52'N) is located in the Xilingol League in the central Inner Mongolia Autonomous Region to the north of China (Fig 1). This grassland has a total area of 193,000 km^2^, a usable grassland area of 180,000 km^2^ and can be divided into five main types: typical grasslands, desert grasslands, meadow grasslands, sandy grasslands and others. The study area has a northern temperate continental climate characterized by strong winds, as well as mostly arid conditions and cold temperatures. The mean annual temperature is 0–3°C, and the multi-year mean precipitation is 295 mm. Precipitation gradually decreases from the southeast to the northwest, and is mostly concentrated in July, August and September.

### Data sources and processing

Remote sensing data used in this study—the 500-m×500-m, 8-day, composite land surface reflectance product (MOD09A1) from 2001–2013 for the Xilingol League—were obtained from the Land Processes Distributed Active Archive Center of the United States Geological Survey (https://lpdaac.usgs.gov/). Normalized difference vegetation index (NDVI) data were obtained by calculation.

Meteorological data (including the monthly mean temperature (°C), monthly precipitation (mm) and sunshine duration (h) from 2001–2013) were obtained from nine national standard meteorological stations of the China Meteorological Administration, namely, the East Ujimqin Banner, Erenhot, Naranbulag (Abag Banner), Abag Banner, Sonid Left Banner, Jurh (Sonid Right Banner), West Ujimqin Banner, Xilinhot and Duolun Meteorological Stations (Fig 1). Raster meteorological data are required for models of the vegetation NPP, and they were obtained by Kriging interpolation of data from the nine meteorological stations, using an inverse distance-weighted routine of the open geographic information system (GIS) software SAGA GIS version 2.2.7 [34]. Pixel size and projection type of the resulting raster data were consistent with the NDVI data.

Measured NPP was based on 46 monitoring stations within the Xilingol in July of 2011, as shown in Fig 1. Three pairs of 0.5m×0.5m sample plots were established at each monitoring station. Plant species composition, height and coverage were measured in all plots, and then in one of each pair, all vegetation was harvested (clipped) at the soil surface and dried at 75°C for 48 h prior to weighing. Aboveground biomass (AGB) was estimated by averaging the biomass of the three harvested plots. Belowground biomass (BGB) was estimated using root augers of 8.9 cm diameter to a depth of 20 cm. Total biomass was calculated as the sum of AGB and BGB. A coefficient 0.475 was used to convert biomass (g·m^–2^) to NPP (gC·m^–2^·a^–1^) [35].

We also collated data on livestock numbers in the nine counties in the Xilingol League between 2001 and 2013, using the Statistical Yearbooks of the Xilingol League of 2002–2014 [36]. Our analysis included sheep and large livestock (cattle and horses). According to the National Bureau of Statistics of the People's Republic of China, since 2008 all recorded numbers of horses, cows and sheep are based on sampling survey. The method used by Li, 2007 [37] was used to convert the data to the unit of a standard sheep (1 head of large livestock (cattle or horse) = 5 standard sheep).

### NPP estimation model

We applied the CASA model first developed by Potter et al. (1993) [18] and Field et al. (1995) [21] which is based on light use efficiency model. A modified version was developed by Zhu et al [38], and was used in this study. The main equations for estimating NPP are as follows:

NPP(x,t)=APAR(x,t)×ε(x,t) (1)

APAR(x,t)=SOL(x,t)×FPAR(x,t)×0.5 (2)

where *SOL(x*,*t)* represents the total solar radiation at pixel *x* in month t (MJ·m^-2^·month^-1^), and *FPAR(x*,*t)* represents the fraction of the incident PAR absorbed by the vegetation. The value of 0.5 represents the fraction of total solar radiation that can be used by vegetation (0.38–0.71mm).

FPAR can be expressed based on relationships between FPAR and NDVI as well as Simple Ratio (SR), which are calculated from Eqs 3 to 6:

$$FPAR\left(x,t\right)=\left[FPAR{\left(x,t\right)}_{SR}+FPAR{\left(x,t\right)}_{NDVI}\right]/2$$ (3)

$$FPAR{\left(x,t\right)}_{NDVI}=\frac{\left(NDVI\left(x,t\right)-NDVI_{min}\right)}{\left(NDVI_{i,max}-NDVI_{i,min}\right)}\times \left(FPAR_{max}-FPAR_{min}\right)+FPAR_{min}$$ (4)

$$FPAR{\left(x,t\right)}_{SR}=\frac{\left(SR\left(x,t\right)-SR_{min}\right)}{\left(SR_{max}-SR_{min}\right)}\times \left(FPAR_{max}-FPAR_{min}\right)+FPAR_{min}$$ (5)

SR(x,t)=[1+NDVI(x,t)]/[1–NDVI(x,t)] (6)

Where *NDVI*_*i*,*max*_ and *NDVI*_*i*,*min*_ are maximum and minimum values of NDVI, and correspond to different plant types (obtained from Land Cover Products of China, Environmental and Ecological Science Data Center for West China, National Natural Science Foundation of China) (http://westdc.westgis.ac.cn) [39]. *FPAR*_*min*_ was 0.001 and *FPAR*_*max*_ was 0.95, both of which are independent of vegetation type. *SR*_*i*,*max*_ and *SR*_*i*,*min*_ represent the 95% and 5% of NDVI respectively, for the different vegetation types.

The algorithm for light use efficiency can be expressed as follows:

$$\varepsilon \left(x,t\right)=T_{\varepsilon 1}\left(x,t\right)\times T_{\varepsilon 2}\left(x,t\right)\times W_{\varepsilon }\left(x,t\right)\times \varepsilon_{max}$$ (7)

where *T*_*ε*1_(*x*,*t*) and *T*_*ε*2_(*x*,*t*) are the temperature stress coefficients, which reflect the reduction of light-use efficiency caused by temperature [21]. *W*_*ε*_(*x*,*t*) is a moisture stress coefficient which is derived from the reduction in light use efficiency caused by moisture stress [21], and *ε*_*max*_ is the maximum light use efficiency under ideal conditions (which can be set to different constant parameters for different vegetation types). The value of *ε*_*max*_ for grassland is 0.542 gC·MJ^-1^ and for shrubs is 0.429 gC·MJ^-1^ in this study. *ε*_*max*_ is clearly an important parameter when applying the CASE model. The values used in this manuscript were previously tested by Zhu,et al [38], using a modified, least squares function and based on NOAA/AVHRR remote sensing and field-observed NPP. These *ε*_*max*_ values have been used to estimate NPP at different spatial scales—including the whole of China, Inner Mongolia, and Xilingol grasslands. All the above estimates of NPP have been shown to be reliable [29,38,40,41]. A more detailed description of this algorithm is available [38,42].

### Yearly and seasonal NPP

Yearly and seasonal NPP are presented to illustrate spatio-temporal variation in our study area. Yearly NPP was calculated as the sum of NPP from January to December. However, in the CASE model, as shown in Eqs 2 to 7, the temperature stress coefficient (that depends on air temperature) and moisture stress coefficient (that depends on precipitation) dictate that in winter (from December to February of the following year), NPP was effectively zero. Similarly, NDVI based on remote sensing, was positive only after plant emergence in early spring, remaining so until late autumn. Consequently, NPP were not estimated (assumed zero) for the months of December to the following February. Annual NPP in this study is thus equivalent to growing season NPP.

### Method for verifying NPP estimation results

We used R version 3.4.0 [43] and R Studio version 1.0.143 [44] for statistical analyses, including linear regression and coefficients T-text, F-test, bias, time series autocorrelation analysis [45], the determination coefficient (R^2^) and the root-mean-square error (RMSE) of the linear fit (goodness-of-fit):

$$RMSE=\sqrt{\frac{\sum_{i=1}^{n}{\left(P_{i}-O_{i}\right)}^{2}}{n}}$$ (8)

where *P*_*i*_ and *O*_*i*_ represent the estimated and observed values, respectively (i = 1, 2,…, n, where n represents the number of samples).

### Correlation and partial correlation analyses between the NPP and climate

Pixel-based correlation coefficients and partial correlation coefficients between derived NPP and temperature and precipitation data were calculated at annual and seasonal scales to assess correlations between NPP and temperature and precipitation.

Correlation coefficients (R) were calculated as follows:

$$R_{}=\frac{\sum_{i=1}^{n}\left[\left(x_{i}-\bar{x})(y_{i}-\bar{y}\right)\right]}{\sqrt{\sum_{i=1}^{n}{\left(x_{i}-\bar{x}\right)}^{2}\sum_{i=1}^{n}{\left(y_{i}-\bar{y}\right)}^{2}}}$$ (9)

where *x* and *y* represent two variables; $\bar{x}$ and $\bar{y}$ represent the mean values of *x* and *y*, respectively; *R*_*xy*_ represents the correlation coefficient between *x* and *y*; and *n* represents the number of samples.

We used partial correlation (R_p_) analysis where:

$$R_{p}=\frac{r_{12}-r_{13}r_{23}}{\sqrt{\left(1-r_{13}^{2}\right)\left(1-r_{23}^{2}\right)}}$$ (10)

and *r*_*12*_, *r*_*13*_ and *r*_*23*_ represent the correlation coefficients between variables *X*_*1*_ and *X*_*2*_, between variables *X*_*1*_ and *X*_*3*_ and between variables *X*_*2*_ and *X*_3_, respectively. *R*_*p*_ represents the partial correlation coefficient between *X*_*1*_ and *X*_*2*_ when *X*_*3*_ is the control variable.

The partial correlation equation above was used to calculate partial correlation coefficients between NPP and temperature when precipitation was the control variable, as well as partial correlation coefficients between NPP and precipitation when temperature was the control variable.

## Results

### Verification of NPP estimates

**Fig 2:**
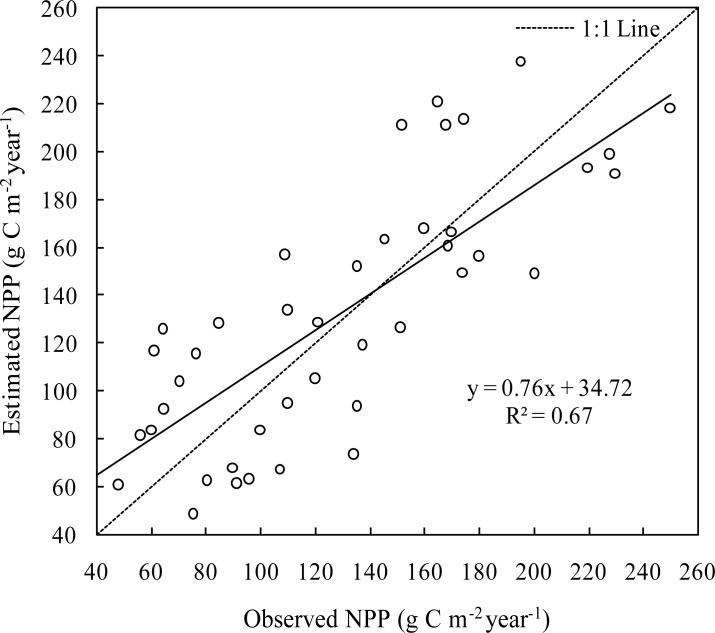
Correlation between estimated and observed NPP. The slope of the linear regression equation is 0.76 (p < 0.001, n = 46, significantly different from 1) while the intercept was 34.7 (p < 0.01, n = 46, significantly different from 0). The calculated F-statistic was 87.4 (p< 0.0001, n = 46) with a correlation coefficient (R^2^) of 0.67 (P<0.0001). Calculated bias of observation and estimation NPP is -0.11 g C·m^-2^·year^-1^ while the RMSE was 35 g C·m^-2^·year^-1^.

Monitoring data from 46 monitoring stations were compared with simulated NPP for 2011 (Fig 2). Estimated NPP was the sum of NPP from January to July. Correlations between simulated and measured values were based on geographic coordinates of each station (Fig 1).

Statistical data indicate a reasonably strong, linear relationship between estimated and observed values. On this basis, we used estimates from the CASA model to further analyse spatiotemporal changes in NPP, as well as assess relationships to climate.

### Inter-annual changes in the NPP, precipitation and temperature

**Fig 3:**
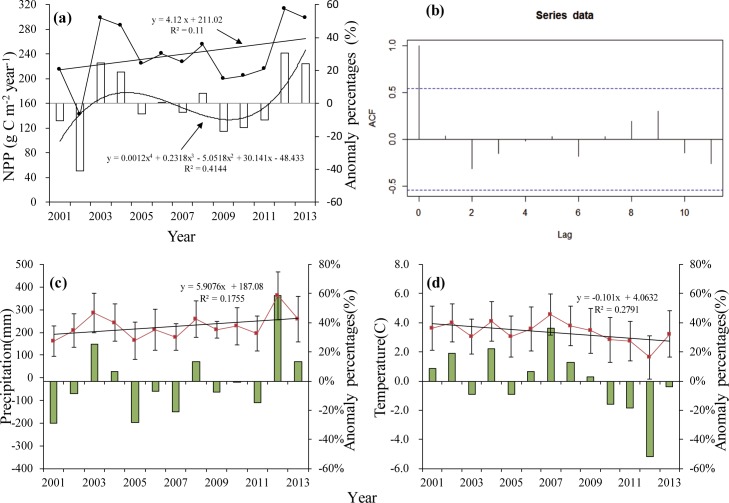
Changes in the NPP, precipitation and temperature in the study area between 2001 and 2013. **(a)** Mean NPP (dotted line), linear regression of the mean NPP (straight line), difference between annual NPP and long-term NPP (histogram), and linear regression of the difference between annual NPP and long-term NPP (straight line). **(b)** Time series auto-correlation function (acf) of NPP from 2001 to 2013. **(c)** Mean precipitation (dotted line), linear regression of the mean precipitation (straight line) and difference between annual and long-term precipitation (histogram). **(d)** Mean temperature (dotted line), linear regression of mean temperature and difference between annual and long-term temperature (histogram).

Fig 3(A) shows annual mean NPP, and NPP anomalies, for the vegetation in the Xilingol League between 2001 and 2013. Mean NPP varied between 141 and 313 g C·m^-2^·year^-1^, with a 13-year mean of 240 g C·m^-2^·year^-1^. Total NPP exhibited an increasing but insignificant (r^2^ = 0.11, p = 0.274) trend with time. Between 2001 and 2013, differences between annual NPP and long-term (13 year) means exhibited a sinusoidal shape. Time series autocorrelation analysis Fig 3(B) shows interannual NPP were not significantly related.

Precipitation generally increased during the study period and was greatest (361 mm) in 2012, some 58% greater than the multi-year mean (Fig 3(C)). Precipitation totals for 2001 and 2005 were relatively low, some 29% and 28% less than multi-year mean precipitation, respectively. Mean annual temperatures varied between 4.58°C in 2007 and 1.62°C in 2012, with a period mean of 3.36°C (Fig 3(D)). Overall, mean annual temperature declined during the study period (r^2^ = 0.279, p = 0.06).

**Fig 4:**
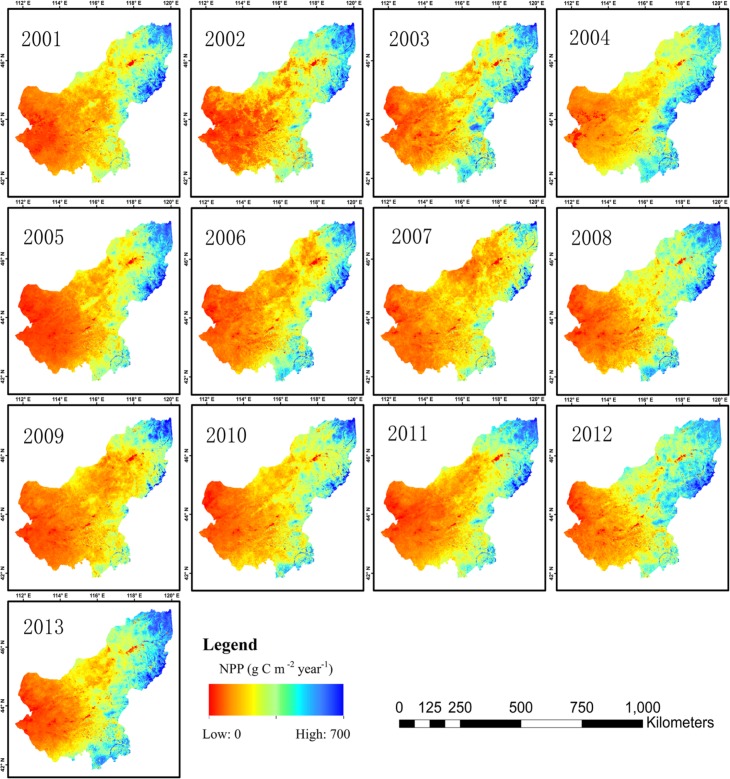
Spatial distribution of the annual total NPP between 2001 and 2013.

Fig 4 shows the spatial distribution of NPP in the Xilingol League between 2001 and 2013. NPP in most regions was <500 g C·m^-2^·year^-1^. NPP in Abag Banner, Sonid Left Banner and Jurh in the western Xilingol League was between 100 and 300 g C·m^-2^·year^-1^, but was < 100 g C·m^-2^·year^-1^ in some regions of Erenhot. NPP significantly increased from west to east, and in West Ujimqin Banner and East Ujimqin Banner, NPP was between 300 and 700 g C·m^-2^·year^-1^.

### Analysis of relationships among NPP, precipitation and temperature

Temperature and precipitation exert critical control of vegetation NPP in most ecosystems [10,25,26]. Temperature and precipitation were used to calculate NPP via the CASE model. We note that Hashimoto et al. [46] showed the CASE model to be sensitive to variation in shortwave radiation and NDVI.

NPP, precipitation and temperature for each county were averaged according to season. Correlations between precipitation and temperature and NPP are shown in Table 1.

**Table 1:** Correlation coefficients (r) between annual and seasonal NPP and climate variables.

Station Names of stations	Correlation coefficients between NPP and climate variables							
	Annual NPP		Spring NPP		Summer NPP		Autumn NPP	
	AP	AMT	SpP	SpMT	SuP	SuMT	AuP	AuMT
East Ujimqin Banner	0.594*	-0.126	0.632*	0.560*	0.531	-0.3	0.826**	0.073
Erenhot	0.135	0.131	0.303	0.777**	0.281	-0.336	0.558*	0.137
Naranbulag (Abag Banner)	0.828**	-0.452	0.618*	0.654*	0.803**	-0.174	0.732**	-0.079
Abag Banner	0.775**	-0.383	0.657*	0.610*	0.699**	-0.416	0.761**	-0.287
Sonid Left Banner	-0.042	0.402	0.54	0.808**	0.094	-0.255	0.337	0.326
Jurh (Sonid Right Banner)	0.690**	-0.126	0.582*	0.759**	0.665*	-0.054	0.605*	0.079
West Ujimqin Banner	0.465	-0.107	0.382	0.762**	0.534	-0.251	0.275	0.267
Xilinhot	0.682*	-0.304	0.453	0.751**	0.53	-0.525	0.738**	-0.089
Duolun	0.778**	-0.284	0.151	0.701**	0.646*	-0.467	0.748**	-0.331

AP = annual mean precipitation; AMT = annual mean temperature; SpP = spring precipitation; SpMT = spring mean temperature; SuP = summer precipitation; SuMT = summer mean temperature; AuP = autumn precipitation; AuMT = autumn mean temperature.

* indicates a significant correlation at the 0.05 level (two-tailed).

** indicates a significant correlation at the 0.01 level (two-tailed).

Annual NPP was mostly positively correlated with annual precipitation (Table 1). Strong correlations were observed for East Ujimqin Banner, Naranbulag (Abag Banner), Abag Banner, Jurh (Sonid Right Banner), Xilinhot and Duolun. Conversely, NPP was generally negatively correlated with mean temperature, albeit not significantly. These overall patterns were not always borne out at regional and seasonal scales. For example, Spring temperatures were much more influential of Spring NPP than annual temperatures were of annual NPP. Similarly, Summer precipitation was particularly important to summer NPP (Table 1). There were regional exceptions. NPP for Erenhot, Sonid Left Banner and West Ujimqin Banner were seldom well predicted by either precipitation or temperature and only Spring temperatures had significant predictive power for NPP in these counties.

### Spatial relationships between the annual NPP and precipitation and temperature

**Fig 5:**
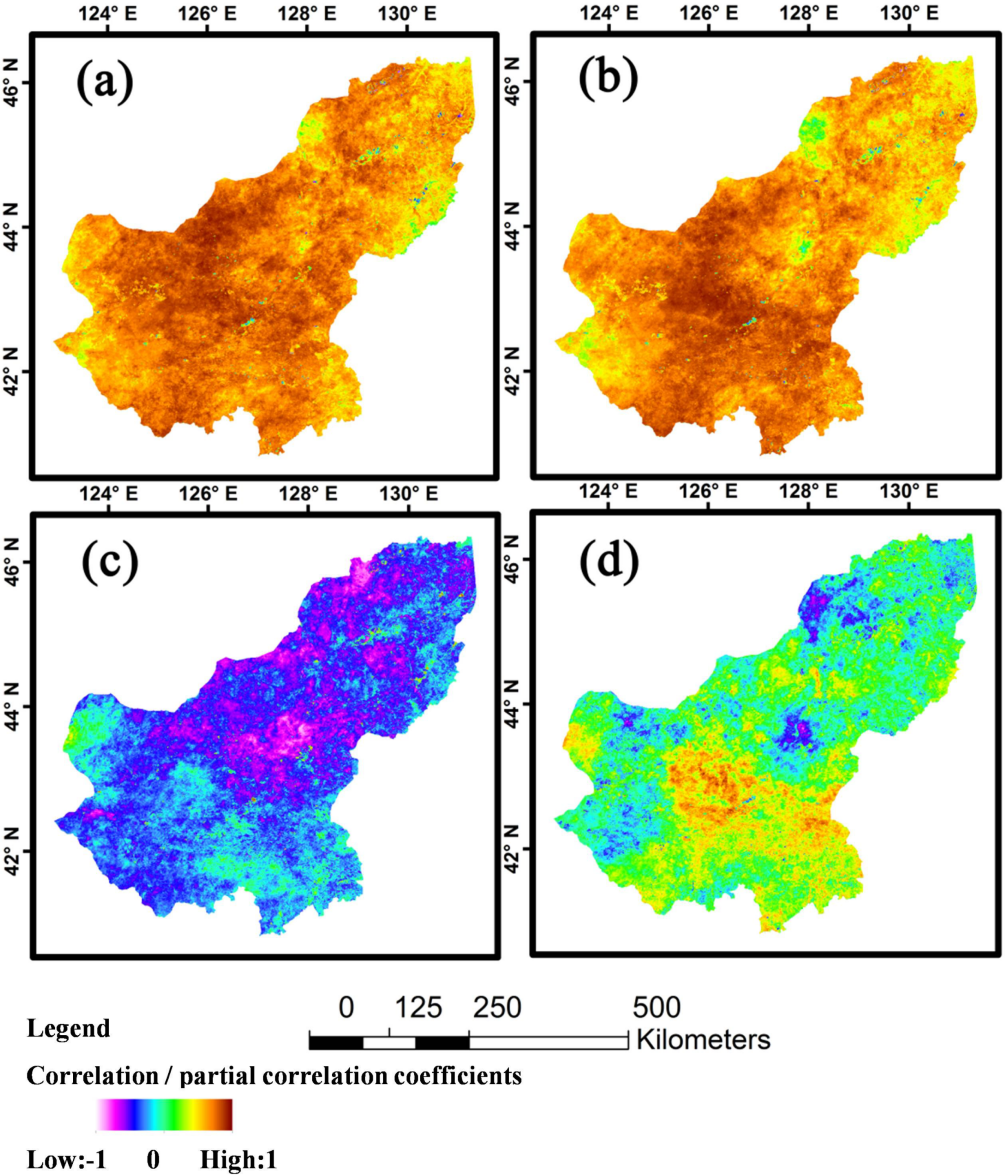
Correlation coefficients and partial correlation coefficients between annual mean NPP and climate variables. **(a)** Correlation analysis between NPP and precipitation. **(b)** Partial correlation analysis between NPP and precipitation. **(c)** Correlation analysis between NPP and temperature. **(d)** Partial correlation analysis between NPP and temperature.

To further analyse spatial relationships between NPP and precipitation and temperature, we calculated correlation coefficients (R, Eq 4) and partial correlation coefficients (R_p_, Eq 5) between the annual NPP of each pixel of the study area and annual precipitation and annual mean temperature between 2001 and 2013 (Fig 5).

Using temperature as the control factor (Fig 5(A)), there were no significant spatial or quantitative differences between R and R_p_ for the relationship between NPP and precipitation. In most regions in the Xilingol League, NPP was significantly positively correlated with precipitation, with R ranging from 0.6 to 1.0. NPP was negatively correlated with precipitation in only 0.32% of regions in the Xilingol League, with a mean correlation coefficient of 0.34. After the effect of the temperature was removed (R_p_, Fig 5(B)), there was almost no change in the relationship between the precipitation and the NPP in the study area.

Fig 5(C) and 5(D) show negative correlations in most regions (99%) between NPP and temperature, before the removal of the precipitation effect. R ranged from -0.8 to 0. In around 1% of the regions in the study area, NPP was positively (but not significantly) correlated with annual mean temperature. After the effect of the precipitation was removed, 77% of regions showed a positive partial correlation between NPP and annual temperature (Fig 5(D)).

### Pixel-scale seasonal relationships between NPP and precipitation and temperature

**Fig 6:**
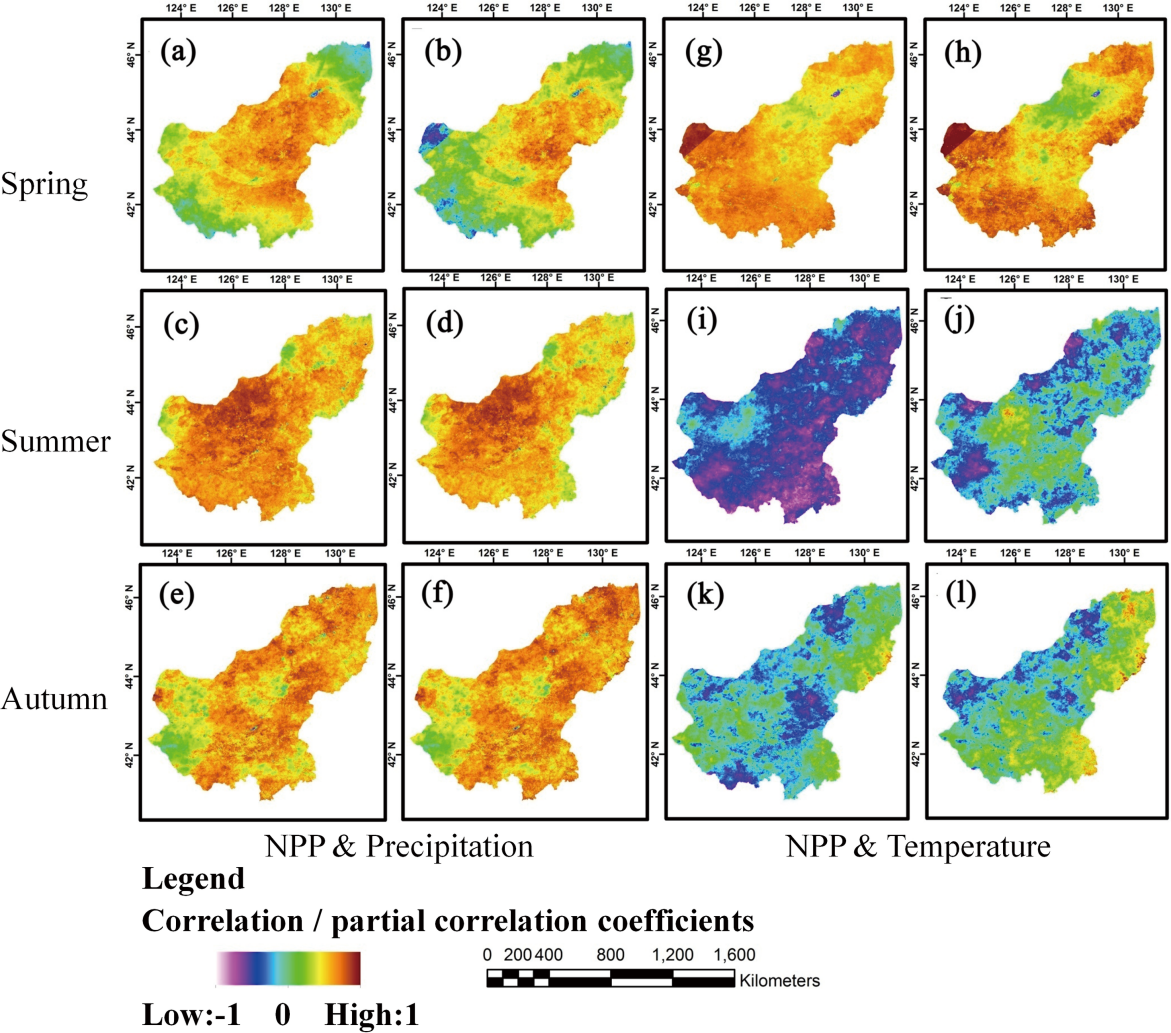
Analysis of correlations and partial correlations between NPP and climate variables across seasons. **(a)**, **(c)**, **(e)** Correlation analysis of NPP and precipitation. **(b)**, **(d)**, **(f)** Partial correlation analysis of NPP and precipitation. **(g)**, **(i)**, **(k)** Correlation analysis of NPP and temperature. **(h)**, **(j)**, **(l)** Partial correlation analysis of NPP and temperature.

Owing to snow cover and lack of growth in Winter, our study was restricted to Spring, Summer and Autumn. For an even greater level of spatial detail, we calculated R and R_p_ for relationships between NPP and climatic variables (precipitation and temperature) from 2001 to 2013 at the pixel scale. Fig 6(A)–6(F) shows that R and R_p_ for NPP and precipitation changed little across spring, summer and autumn. NPP was mostly positively correlated with precipitation. Negative relationships between NPP and precipitation were only significant in Spring, mostly after the effect of temperature was removed, and were largely confined to the south-west portion (Fig 6(A) and 6(B)).

Temperature effects on NPP were more variable (Fig 6(G)–6(L)). Mostly positive relationships in Spring were replaced by negative or neutral relationships in Summer (especially) and Autumn. High summer temperatures are clearly detrimental to NPP for much of the study area. Most of the temperature effects were strongly mitigated by rainfall (contrast R and R_p_ in summer).

## Discussion

Our data for NPP in the study region are quantitatively similar to those reported using other approaches. For example, Li and Ji [47] simulated NPP of grasslands throughout Inner Mongolia using the AVIMia model (Atmosphere-Vegetation Interaction Model and an impact assessment) and found that the multi-year mean NPP ranged from 223 to 315 g C·m^-2^·year^-1^. Results of Zhu et al. [40] suggested that NPP of meadows, plain grasslands and desert grasslands was 383 g C·m^-2^·year^-1^, 226 g C·m^-2^·year^-1^ and 103 g C·m^-2^·year^-1^, respectively. Our data suggest NPP of the Xilingol Grassland increased slightly over the 13-year study period. Zhang [48] analysed the dynamics of Xilingol Grassland in the growing season (April–October) between 2003 and 2012 using NDVI data and noted a similar trend, consistent with results obtained by Jiang et al. [49] through experimentation at fixed locations. The analysis provided here, via integration of validated modelling with remote sensing, offers opportunity to extend such studies to other grassland regions in China, and globally.

Precipitation and temperature effects on grassland productivity have been reasonably well studied [50,51], with the former being especially significant in arid regions [9,52]. It is particularly important to understand the potential influence of climate change on ecosystems within this area, given its large population [53]. Meta-analysis showed that reductions in precipitation have significant influence on aboveground NPP (ANPP). Conversely, increased precipitation can promote ANPP, belowground NPP (GNPP) and NPP [50]. The results of this study support such a general interpretation; positive correlations between annual NPP and precipitation in most regions of the study area demonstrate the strength of control. In addition, negative correlations between temperature and NPP were mostly conditional upon precipitation. These effects are seen most clearly via the differences between R and R_p_ for the relationships of precipitation and temperature to NPP (Fig 5(A) and 5(B) and Fig 6(A)–6(F)). Moreover, most research shows that seasonal precipitation has large impact on the NPP of grassland ecosystems worldwide [54–56]. Our results (see S1(A), S1(C), S1(E) and S1(G) Fig) show that removing temperature had no effect on the distribution ranges of correlation coefficients for the relationship between NPP and precipitation. In simple terms, temperature had no impact on the relationship between precipitation and NPP, whether annual or seasonal (spring, summer and autumn). Conversely, temperature effects were clearly precipitation-dependent. For example, at the annual scale, when precipitation effects were included, NPP in the study area was negatively correlated with temperature in 99% of regions (Fig 5(C) and S1(B) Fig). However, when precipitation was removed (Fig 5(D) and S1(B) Fig), there was a positive correlation in 77% of regions. This pattern is clearly related to the biology of plant growth. Growth is stimulated by increased temperatures when water is readily available [57,58]. Under drought conditions, high temperatures can severely reduce growth [59]. Consequently, correlations and partial correlations between NPP and temperature were consistently positive in Spring (Fig 6(G) and 6(H) and S1(D) Fig) and mainly negative in Summer (Fig 6(I) and 6(J) and S1(F) Fig) and autumn (Fig 6(K) and 6(L) and S1(H) Fig).

As is commonly recognized, aside from precipitation and temperature, NPP is also subject to influence by other environmental/climate factors and human activities [60,61]. For example, Zhao [62] found that high temperatures and droughts between 2000 and 2009 were primary causes of reduced global NPP, and Han et al. [63] found that precipitation and temperature contributed almost 60% of the variation in the total biomass. Nonetheless, human activities remain one of the main reported causes of grassland degradation [64–66]. Our assessment is that numbers of grazing livestock declined between 2001 and 2013, and were not significantly related to NPP (S2 Fig). In contrast, using a NDVI-based method alone, Li et al. [67] concluded that human activities (grazing) were the main driver of changes in the vegetation between 1981 and 2006.

## Supporting information

S1 Fig Histograms of the correlation coefficients (dark green) and partial correlation coefficients (light yellow) between the NPP and the precipitation and temperature. **(a)** Annual NPP and precipitation. **(b)** Annual NPP and temperature. **(c)** NPP and precipitation in spring. **(d)** NPP and temperature in spring. **(e)** NPP and precipitation in summer. **(f)** NPP and temperature in summer. **(g)** NPP and precipitation in autumn. **(h)** NPP and temperature in autumn.
(EPS)

S2 Fig (a) Number of livestock (solid red line) and change in the NPP (broken blue line) and (b) the correlation between the number of livestock and the change in the NPP.
(EPS)
